# Dietary and the Risk of Sporadic Colorectal Cancer in China: A Case-control Study

**Published:** 2018-09

**Authors:** Wenfei WANG, Zhaogang DONG, Xin ZHANG, Wei LI, Peilong LI, Xiaoyang CHEN

**Affiliations:** 1. Humanistic Medicine Research Center, Shandong University, 107 Wenhua Xi Road, Jinan 250012, China; 2. Qilu Hospital, Shandong University, 107 Wenhua Xi Road, Jinan 250012, China

**Keywords:** Colorectal neoplasms, Diet, Risk factors

## Abstract

**Background:**

High-fat diets have been considered a risk factor for sporadic colorectal cancer (CRC) in Western countries. However, data for this phenomenon are lacking in China. The purpose of this study was to comprehensively evaluate the association between diet and the risk for sporadic CRC in Shandong Province, China.

**Methods:**

In this case-control study, 317 patients with sporadic CRC and 317 controls were collected in Shandong Province, China. All the samples were collected on the basis of rigorous screening criteria. The data were analyzed using a χ^2^ test, univariate or multivariate conditional logistic regression, and stratified analysis.

**Results:**

Multivariate logistic regression showed that the following are risk factors for sporadic CRC (all *P*<0.05): consumption of pork, fried food or barbecued meat; high Body Mass Index (BMI); alcohol abuse; psychosis; and the presence of a factory causing pollution near the home. Moreover, univariate analysis revealed the following qualities were also positively associated with CRC (all *P*<0.05): intake of animal oil, consuming brawn and kipper, smoking, exhibiting frequent anger, and poor sleep quality. Eating fresh fruit was inversely correlated with the incidence of CRC (*P*=0.012). Further stratified analysis demonstrated that BMI and the consumption of fried food, barbecued meat, or garlic were correlated with colon cancer. However, alcohol abuse and psychosis were related to an increased risk for rectal cancer.

**Conclusion:**

Dietary factors are related to sporadic CRC in Shandong Province. Future interventions should focus on reducing the related risk factors while advocating for practice of the protective factors.

## Introduction

Colorectal cancer (CRC) is one of the most common malignant tumors in the world and the fifth leading cause of cancer death in China (1). Data from the Shandong Center for Disease Control and Prevention (SDCDC) reported that out of 20100 new cases of CRC, 8940 deaths resulted (2). Two-thirds of these cases are sporadic CRC, the type most commonly seen in clinical practice (3). Local environment and individual’s genetic makeup may have impact on sporadic CRC. (3). To improve patient survival in CRC, it is necessary to pay more attention to potential measures for disease prevention and control.

Sporadic CRC is cancers that arise from the colorectum with no known contribution from either germline mutations or a significant family history of cancer or inflammatory bowel disease (3). Interestingly, the same mutations occur in both inherited and sporadic CRC to a significant extent (4). Some of the same factors are involved in somatic or acquired alterations in sporadic CRC. Colorectal tumorigenesis is a multi-step process, and certain dietary habits have been shown to strongly contribute to sporadic CRC. However, there are regional differences, and the evidence is inconsistent (5). Red meat consumption is associated with an increased risk for sporadic CRC in Western countries, such as France (6) and Australia (7). However, meat intake did not differ significantly between 265 CRC cases and 252 controls in China (8). In fact, the consumption of barbecued meat and fried meat is relatively high in the Shandong population, but studies concerning the association between processed meat and CRC in Shandong Province are rare. Garlic is also consumed as a food additive throughout the world and is very popular in Shandong Province. However, whether garlic consumption plays a role in CRC is uncertain. Garlic consumption reduced the incidence of CRC (9). In another study, however, no association between garlic intake and risk of CRC was found (10). Additionally, Body Mass Index (BMI), smoking status, and lifestyle have significant impacts on dietary intake; however, these results are controversial (11, 12). Taken together, the association between diet and the risk for CRC is a contentious subject. Thus, the role of potential factors that may contribute to CRC in our region should be further investigated.

To further understand the association between dietary and sporadic CRC, we conducted a retrospective case-control study in Shandong Province, China. In addition, the potential predictors for colon cancer might be different from that of rectal cancer, we also performed a stratification analysis between colon cancer and rectal cancer.

## Materials and Methods

### Study Population

The present study using a case-control design for CRC was conducted in Shandong Province, China. Cases were enrolled at Qilu Hospital of Shandong University. The enrollment started in May 2007 and ended in Aug 2010. The survey was conducted from July 2015 to Aug 2016 to ensure that all subjects have more than 5 years of follow-up records. Cases were age- and gender-matched on a 1:1 (case-control) ratio in the same time frame. The controls were recruited from the healthy population residing in the same areas as the cases for more than 5 years.

This study was approved by the Ethics Committee of Qilu Hospital of Shandong University. Written informed consent was obtained from all subjects.

The inclusion criteria for cases were as follows: newly diagnosed independently by two expert gastrointestinal pathologists, aged 33–83 yr old, extended living in Shandong Province, and able to complete the interview independently. We excluded 263 participants for the following reasons: (1) They had cancer previously (n=112); (2) Incomplete or inconsistent data (n=46); (3) Data were not representative of the cohort (n=68); and (4) Familial adenomatous or hereditary nonpolyposis colon cancer (n=37). Consequently, 317 CRC patients (215 colon cancer and 102 rectal cancer) were recruited into this study.

The inclusion criteria for controls were as follows: (1) Matched for gender and age at enrollment (within 5 yr), (2) Free of cancer and other disorders of gastrointestinal tract, and (3) No family history of CRC in a first-degree relative. Overall, 241 controls were ineligible because of incorrect information. Overall, 317 controls were included in this study.

### Data collection

Data were obtained from each participant via personal interviews using structured questionnaires administered by trained investigators. Cases were asked to complete a questionnaire consisting of two parts: their dietary habits and diet-related factors including environment, lifestyle, and psychosocial status before diagnosis. Baseline characteristics including age, gender, education level, vocation, and marital status were also recorded. Controls were asked the same information before the interview date. The interview took approximately one hour.

### Quality control

The measures for quality control were as follows: To make unified guidelines of investigation and survey methods, investigators were strictly trained, and the same investigator recorded responses for the matched case and control. Moreover, the reliability and validity of used questionnaire are strictly controlled. The questionnaire design is complete, accurate and incompatible. The data are strictly audited and the selected questions are representative. A proper time and site were chosen and make sure that the respondents cooperate actively, conscientiously and objectively answer the questions. In addition, the reliability and validity are evaluated by using reliability coefficient and validity coefficient, respectively. Finally, the questionnaire was performed by at least two investigators simultaneously and the completed questionnaire and data were carefully reviewed and checked for accuracy.

### Statistical analysis

All data were analyzed by SPSS software (ver. 13.0, SPSS Inc., Chicago, IL). All statistical tests were two-sided, and *P*<0.05 was considered statistically significant. Differences in the distribution of baseline characteristics and potential factors were compared between cases and controls using a χ^2^ test. All variables were analyzed with univariate conditional logistic regression, and those with a significant difference between cases and controls (*P*<0.05) were entered into the multivariate conditional logistic regression analysis. The odds ratio (OR) and 95% confidence intervals (95% CI) were calculated to describe the association between each variable and CRC, respectively. Stratified analysis was used to assess the predictors for colon cancer and rectal cancer.

## Results

### The univariate logistic analysis of risk and protective factors in sporadic CRC

Table 1 presents the baseline characteristics of CRC cases and control subjects. The distribution of age, gender, education level, vocation, and marital status did not significantly differ between CRC cases and control subjects (all *P*>0.05). Compared with control subjects, CRC subjects were more likely to have high BMI (≥25 kg/m^2^), or to consume more pork, animal oil, fried food, barbecued meat, and smoked food or brawn and kipper (all *P*<0.05) but exhibited less consumption of fresh vegetables (dietary fibers), fresh fruit, and garlic (all *P*<0.05) (Tables 2 and 3).

**Table 1:** The distribution of baseline characteristics

***Demographics***		***CRC***	***Control***	***χ^2^ value***	**P-*value***
Age				0.007	0.932
	< 60	216	215		
	≥ 60	101	102		
Gender				0.006	0.936
	Male	145	146		
	Female	172	171		
Education level				0.397	0.941
	Illiteracy	84	88		
	Junior school	66	60		
	Junior high school	33	34		
	High school or above	134	135		
Vocation					
	Farmer	173	176	1.044	0.593
	Worker	93	83		
	The rest	51	58		
Marital status				0.016	0.992
	Married	261	262		
	Divorced	21	21		
	Widowed	35	34		

**Table 2:** Univariate logistic analysis of risk factors

***Factors***	***OR***	***95% CI***	**P-*value***
BMI (kg/m^2^)	2.909	2.094∼4.040	<0.001
Pasta intake	1.272	0.931∼1.739	0.131
Frequencies of pork meat consumption	1.865	1.528∼2.277	<0.001
Frequencies of animal oil consumption	1.270	1.023∼1.575	0.030
Frequencies of fried food consumption	3.310	2.637∼4.154	<0.001
Frequencies of barbecued meat consumption	2.863	2.322∼3.530	<0.001
Frequencies of smoking food consumption	1.517	1.117∼2.060	0.008
Frequencies of brawn and kipper consumption	1.287	1.022∼1.620	0.032
Frequencies of fish and shrimp consumption	1.136	0.872∼1.480	0.346
Frequencies of Beans and its products consumption	1.000	0.801∼1.249	1.000
Food temperature	1.397	0.954∼2.046	0.086
Food taste	1.076	0.810∼1.428	0.613
Smoking	1.906	1.321∼2.750	0.001
Alcohol abuse	1.464	1.038∼2.064	0.030
Drinking tea	1.039	0.761∼1.418	0.812
Psychosis	2.299	1.612∼3.279	<0.001
The factory that causes pollution around home	3.828	2.735∼5.358	<0.001
Often angry	1.671	1.203∼2.321	0.002
Depression	8.631	6.036∼12.343	<0.001
Sleep quality	1.461	1.014∼2.107	0.042

**Table 3:** Univariate logistic analysis of protective factors

***Factors***	***OR***	***95% CI***	**P-*value***
Frequencies of egg and its products consumption	0.802	0.629∼1.022	0.074
Frequencies of milk and its products consumption	0.759	0.559∼1.030	0.077
Fresh vegetables (dietary fibers)	0.350	0.285∼0.429	<0.001
Frequencies of fresh fruit consumption	0.788	0.654∼0.949	0.012
Frequencies of garlic consumption	0.497	0.414∼0.596	<0.001
Food hardness	0.844	0.640∼1.114	0.232
Breakfast	0.989	0.734∼1.332	0.939
Physical exercise	0.681	0.478∼0.968	0.032

Additionally, some factors that influenced appetite, such as environment, lifestyle, and psychosocial status, were also considered. Interestingly, lifestyle, for example, “smoking” (*P*=0.001), “alcohol abuse” (*P*=0.030), and “poor sleep quality” (*P*=0.042) were risk factors for CRC. However, more physical exercise (*P*=0.032) was a protective factor. Moreover, psychosocial status including “psychosis” (*P*<0.001), “often angry” (*P*=0.002), and “depression” (*P*<0.001) were positively correlated with CRC. In addition, living near a factory that causes pollution around the home (*P*<0.001) was positively related to CRC. The statistically significant variables (*P*≤0.05) were further evaluated in the multivariate logistic regression.

### The multivariate logistic analysis of risk and protective factors in sporadic CRC

The interaction between factors was also taken into consideration. Multivariable logistic regression models were constructed with stepwise regression analysis. After adjustment for potential confounders, this positive association remained significant for “BMI” (*P*=0.005), “more consumption of pork” (*P*=0.035) or “fried food” (*P*<0.001) or “barbecued meat” (*P*<0.001), “alcohol abuse” (*P*=0.010), “psychosis” (*P*=0.011), “nearby factory causing pollution around the home” (*P*<0.001), and “depression” (*P*<0.001). “Fresh vegetables” (*P*<0.001), “garlic” (*P*<0.001), and “more physical exercise” (*P*<0.001) were significantly related to a decreased risk for CRC (Table 4).

**Table 4:** Multivariate logistic regression analysis of risk factors

***Factors***	***OR***	***95% CI***	**P-*value***
BMI (kg/m^2^)	2.038	1.012∼4.107	0.005
Frequencies of pork meat consumption	1.587	1.044∼2.414	0.035
Frequencies of fried food consumption	3.630	2.275∼5.792	<0.001
Frequencies of barbecued meat consumption	2.621	1.708∼4.021	<0.001
Fresh vegetables (dietary fibers)	0.247	0.162∼0.375	<0.001
Frequencies of garlic consumption	0.499	0.341∼0.732	<0.001
Alcohol abuse	2.390	1.128∼5.062	0.010
Psychosis	3.165	1.417∼7.072	0.011
The factory that causes pollution around home	5.302	2.539∼11.075	<0.001
Depression	13.954	6.487∼30.017	<0.001
Physical exercise	0.232	0.108∼0.499	<0.001

### Stratification analysis by sub-sites of CRC

We performed stratification analysis by the sub-sites of CRC (215 colon cancer cases and 102 rectal cancer cases). After stratified analysis, “fresh vegetables” (dietary fibers), “living near a factory that causes pollution around home”, “depression”, and “physical exercise” were included in the final models of both colon cancer and rectal cancer (all *P*<0.05). “BMI” (*P*=0.033) and “consumption of fried food” (*P*<0.001) or “barbecued meat” (*P*<0.001) or “garlic” (*P*<0.001) were only correlated with colon cancer. “Alcohol abuse” (*P*=0.009) and “psychosis” (*P*=0.019) were related only to an increased risk of rectal cancer (Table 5).

**Table 5:** Stratification analysis of colon and rectal cancer

***Factors***	***Colon cancer***			***Rectal cancer***		
	***OR***	***95% CI***	***P value***	***OR***	***95% CI***	***P-value***
BMI (kg/m^2^)	2.681	1.082∼6.646	0.033	2.033	0.59∼7.01	0.261
Frequencies of pork meat consumption	1.661	0.950∼2.904	0.075	1.336	0.658∼2.713	0.423
Frequencies of fried food consumption	4.522	2.437∼8.393	<0.001	1.610	0.691∼3.748	0.270
Frequencies of barbecued meat consumption	3.242	1.871∼5.618	<0.001	1.845	0.900∼3.786	0.095
Fresh vegetables (dietary fibers)	0.209	0.120∼0.364	<0.001	0.231	0.097∼0.551	0.001
Frequencies of garlic consumption	0.293	0.171∼0.502	<0.001	0.945	0.470∼1.897	0.873
Alcohol abuse	1.885	0.730∼4.865	0.190	7.409	1.641∼33.455	0.009
Psychosis	2.746	0.945∼7.978	0.063	5.167	1.310∼20.386	0.019
The factory that causes pollution around home	4.843	1.856∼12.640	0.001	4.493	1.040∼19.404	0.044
Depression	32.615	10.780∼98.673	<0.001	5.616	1.405∼22.444	0.015
Physical exercise	0.219	0.082∼0.581	0.002	0.060	0.010∼0.348	0.002

## Discussion

Dietary habits have regional variations in China. People in Shandong Province typically have high-fat-diets and like to eat foods processed at high temperatures, such as fried food and barbecued meat. Meat consumption, especially barbecued (7, 13) and fried meat (14, 15), is suspected to be associated with an enhanced risk for CRC. In contrast, the consumption of red baked meat and red pan-fried meat had protective trends for CRC (16). Fried food does not play a relevant role in development of CRC (17). Pork, fried food, and barbecued meat are associated with an elevated risk for CRC. One potential mechanism for this could be that the fat content of meat promotes bile secretion; subsequently, the bile is turned into secondary bile acid, which may play a role in CRC pathogenesis (18). Another mechanism might be that high-temperature meat has high levels of heterocyclic amines and is highly mutagenic, which could lead to DNA damage in colon tissue (14). High-fat-diet affects the composition of the intestinal microbiota, leading to the evolution of CRC (19). Our findings also supported that high-fat-diet could lead to obesity and high BMI, associated with an increased risk of CRC (20). Multivariate analysis showed that it had little relationship with CRC, though univariate analysis revealed it was a risk factor. This discrepancy might be due to the small sample size of this region. Brawn and kipper foods in China are similar as preserved foods, and they were popular foods in Shandong Province in past decades, but people seldom eat this kind of food now because the standard of living is improving.

Some case-control studies have shown protective effects of garlic consumption for colorectal cancer (9, 21). Diallyl disulfide (DADS) is a constituent of garlic that inactivates NFκB and prevents colitis-induced colorectal cancer via inhibition of GSK-3β signaling. (22). Consistent with previous studies, our findings suggested that garlic, as a high yield and commonly consumed food in Shandong Province, was significantly inversely correlated with the risk for colorectal cancer. Evidence from several in vitro studies supports the mechanism for this association, illustrating that garlic induces colon cell apoptosis and arrested the cell cycle (23, 24). In contrast, consumption of garlic was not associated with a reduced CRC risk (11). Potential reasons for this contradiction might be differences in race and eating habits. Increased fruit and vegetable intake could reduce the detrimental effects of high intake of processed meat on CRC risk (25). Our results demonstrated that fresh vegetables (dietary fibers) and fruit were protective factors for CRC.

Pollution is a common problem in China and most countries, and it contributes to a marked increase in CRC (26). China is the most populous low-to-middle-income country in the world. Rapid industrialization and urbanization have been accompanied by incredible changes in lifestyle and environment, combined with air pollution, water pollution, and food contamination. For example, coal burning produces carcinogens and coal ash, which contain radioactive material and heavy metals, such as lead, mercury, and chromium. This is one source of industrial waste. Our results showed that living near a factory that causes pollution around the home was a risk factor for CRC. Uncontrolled environment pollution has directly caused “cancer villages” in China, which is providing important evidence for the link between environmental carcinogens and cancer incidence. (27). In cancer treatment and prevention, China faces many difficulties and challenges. (28). The government must take legal steps instead of administrative measures to protect the environment, such as inhibition of dumping wastewater and increasing drinking water and food safety. Notably, environmental policies should be enforced in both rural and urban areas because factories now mostly favor urban areas.

The rapid development of China’s economy has significantly changed people’s lifestyle. People are feeling increasing pressure and stress. Smoking and alcohol consumption are popular outlets for relieving stress. In China, smoking is still a social custom and giving cigarettes in social interaction has been a sign of respect and friendliness. However, smoking, especially exposure to secondary smoke indoors, increases the risk for CRC (29). Our results indicate that smoking was the risk of CRC. Although cigarette use does not enter the final model, we still call on people to give up smoking. The government has paid attention to this problem, and that smoking has been banned in any public area including schools, the workplace, and public transportation vehicles in several provinces. The relationship between alcohol and CRC remains inconclusive. Alcohol was not associated with an increased risk for CRC (15). Alcohol abuse was related to CRC and increases the mortality (30, 31). Our results were similar to these studies. The underlying mechanism might be related to alcohol metabolism. Alcohol is converted into acetaldehyde by the action of alcohol dehydrogenase, and acetaldehyde can initiate tumors by forming adducts with proteins and DNA (32). Another mechanism could be that alcohol interferes with the intake and absorption of folic acid, leading to increased risk for CRC. Our findings demonstrate that physical exercise and better sleep quality are the protective factors for CRC. Physical exercise could stimulate intestinal peristalsis, which reduces the retention time of food, endogenous secretions (bile acids), and carcinogens in the intestinal tract. This might lower the risk for CRC. Likewise, exercise and better sleep could enhance the body’s immunity to diseases and resist metabolic disorder. Programs need to be established that advocate a healthy lifestyle.

Psychosocial status, such as depression and insomnia, are positively correlated with CRC (33, 34). Our results revealed that psychosis, depression, and often being angry are risks for CRC. The potential mechanism might be that negative psychosocial status results in functional disorder of the body. In the digestive system, the autonomic nervous system might be affected by psychological factors, including psychosis, depression, and often being angry, leading to intestinal peristalsis, the spasm of intestinal vascular smooth muscle, tissue ischemia, and increased capillary permeability. These changes lead to inflammation, anabrosis, and ulceration of intestinal mucosa, which decreases intestinal immunity and increases susceptibility to CRC. Therefore, measures concerning psychological intervention should be taken to relieve people’s stress.

Some significant factors are different between colon cancer and rectal cancer. Interesting, BMI and consumption of fried food or barbecued meat or garlic were only correlated with colon cancer. Alcohol abuse and psychosis were only related to an increased risk for rectal cancer. In the present study, we recruited patients and healthy controls from hospital and community to eliminate the confounding factors. As data were retrospectively collected, one of the limitations is recall bias in our study; another limitation is the number of cases. Therefore, these results should be validated with additional studies.

## Conclusion

Dietary habits are closely related to the risk for sporadic CRC in Shandong Province, and some diet-related factors also have synergistic effects. Future interventions should focus on educating the public about related risk factors and protective factors. These measures may contribute to minimizing the risk for development of sporadic CRC. Further study is required to apply these findings in order to compile appropriate information and effective projects of communication and to formulate intervention strategies.

## Ethical considerations

Ethical issues (Including plagiarism, informed consent, misconduct, data fabrication and/or falsification, double publication and/or submission, redundancy, etc.) have been completely observed by the author.
